# Characterization of Rice Homeobox Genes, *OsHOX22* and *OsHOX24*, and Over-expression of *OsHOX24* in Transgenic *Arabidopsis* Suggest Their Role in Abiotic Stress Response

**DOI:** 10.3389/fpls.2016.00627

**Published:** 2016-05-10

**Authors:** Annapurna Bhattacharjee, Jitendra P. Khurana, Mukesh Jain

**Affiliations:** ^1^National Institute of Plant Genome ResearchNew Delhi, India; ^2^Interdisciplinary Centre for Plant Genomics and Department of Plant Molecular Biology, University of Delhi South CampusNew Delhi, India; ^3^School of Computational and Integrative Sciences, Jawaharlal Nehru UniversityNew Delhi, India

**Keywords:** rice, abiotic stress, homeobox transcription factors, gene expression, DNA binding, protein–protein interaction

## Abstract

Homeobox transcription factors are well known regulators of plant growth and development. In this study, we carried out functional analysis of two candidate stress-responsive HD-ZIP I class homeobox genes from rice, *OsHOX22*, and *OsHOX24*. These genes were highly up-regulated under various abiotic stress conditions at different stages of rice development, including seedling, mature and reproductive stages. The transcript levels of these genes were enhanced significantly in the presence of plant hormones, including abscisic acid (ABA), auxin, salicylic acid, and gibberellic acid. The recombinant full-length and truncated homeobox proteins were found to be localized in the nucleus. Electrophoretic mobility shift assay established the binding of these homeobox proteins with specific DNA sequences, AH1 (CAAT(A/T)ATTG) and AH2 (CAAT(C/G)ATTG). Transactivation assays in yeast revealed the transcriptional activation potential of full-length OsHOX22 and OsHOX24 proteins. Homo- and hetero-dimerization capabilities of these proteins have also been demonstrated. Further, we identified putative novel interacting proteins of OsHOX22 and OsHOX24 via yeast-two hybrid analysis. Over-expression of *OsHOX24* imparted higher sensitivity to stress hormone, ABA, and abiotic stresses in the transgenic *Arabidopsis* plants as revealed by various physiological and phenotypic assays. Microarray analysis revealed differential expression of several stress-responsive genes in transgenic lines as compared to wild-type. Many of these genes were found to be involved in transcriptional regulation and various metabolic pathways. Altogether, our results suggest the possible role of OsHOX22/OsHOX24 homeobox proteins as negative regulators in abiotic stress responses.

## Introduction

Abiotic stress conditions, including drought and salinity, are detrimental for growth and survival of plants. These environmental factors, either singularly or compositely, cause several adverse effects on the productivity of crop plants like rice. However, by adopting biotechnological tools, it is now possible to generate high-yielding stress-tolerant plants. Several TFs have been used as potent tools to engineer abiotic stress tolerance in plants (Hussain et al., 2011). The over-expression of well-characterized abiotic stress-responsive TFs, like dehydration-responsive element binding proteins (DREBs), ABA-responsive element binding proteins (AREBs), no apical meristem (NAM), *Arabidopsis thaliana* activation factor 1/2 (ATAF1/2), cup-shaped cotyledon 2 (CUC2) proteins (NACs), has led to the generation of stress-tolerant transgenic plants without loss in crop yield (Nakashima et al., 2009; Todaka et al., 2015). However, the function of various other TFs in abiotic stress tolerance still remains to be explored.

Homeobox TFs belong to a large gene family and are known to play crucial roles in various aspects of plant development (Gehring et al., 1994; Nam and Nei, 2005). Rice and *Arabidopsis* genomes contain at least 110 homeobox genes each (Jain et al., 2008; Mukherjee et al., 2009). The members of homeobox TF family have been categorized into 14 classes, including HD-ZIP and TALE superclasses (Jain et al., 2008; Mukherjee et al., 2009). The plant-specific HD-ZIP superclass contains highest number of homeobox proteins (48) and is grouped into four subfamilies, HD-ZIP I-IV (Jain et al., 2008; Mukherjee et al., 2009). All HD-ZIP superclass proteins possess HD and leucine-zipper (LZ) domains. Besides this, HD-ZIP II proteins contain ZIBEL and CE motifs, whereas HD-ZIP III proteins contain MEKHLA domain also. In addition, both HD-ZIP III and HD-ZIP IV subfamily proteins harbor START and HD-SAD domains (Mukherjee et al., 2009).

Various HD-ZIP superclass members are known to regulate a variety of developmental processes and abiotic stress responses in plants (Ariel et al., 2007; Harris et al., 2011). The ectopic expression of a HD-ZIP I subfamily member indicated its involvement in leaf development and blue light signaling (Wang et al., 2003), and few members were reported to mediate giberrellin signaling (Dai et al., 2008; Son et al., 2010). HD-ZIP II subfamily members have been implicated in shade avoidance responses in plants (Sessa et al., 2005). HD-ZIP III subfamily members have emerged as vital regulators of apical meristem formation, maintenance of abaxial or adaxial polarity of leaves and embryo, besides vascular development and leaf initiation process in shoot apical meristem region in *Arabidopsis* and rice, respectively (Prigge et al., 2005; Itoh et al., 2008). Further, HD-ZIP IV subfamily members were reported to be crucial determinants of outer cell layer formation of plant organs, leaf rolling process, trichome development and anther cell wall differentiation (Nakamura et al., 2006; Vernoud et al., 2009; Zou et al., 2011). It has been speculated that evolutionary pressure resulted in well orchestrated participation of numerous HD-ZIP subfamily members in developmental regulation of plants (Ariel et al., 2007).

A number of HD-ZIP superclass members have been found to be differentially expressed during abiotic stress conditions in different plant species (Gago et al., 2002; Olsson et al., 2004; Jain et al., 2008; Ni et al., 2008; Bhattacharjee et al., 2015). The involvement of some HD-ZIP I subfamily members have already been reported in modulating abiotic stress responses (Olsson et al., 2004; Song et al., 2012). In recent times, *AtHB12* and *AtHB7* were found to mediate both growth related processes and water stress responses in *Arabidopsis* (Ré et al., 2014). Some studies have demonstrated the role of a few HD-ZIP genes of *Arabidopsis* and rice in abiotic stress tolerance as well (Olsson et al., 2004; Zhang et al., 2012) and they may act as promising candidates for crop improvement (Bhattacharjee and Jain, 2013).

Previously, at least nine of the 14 members of HD-ZIP I family in rice were found to be differentially expressed under abiotic stress conditions (Jain et al., 2008). In the present study, we performed comprehensive expression profiling of two candidate HD-ZIP I family homeobox genes, *OsHOX22* (*LOC_Os04g45810*) and *OsHOX24* (*LOC_Os02g43330*), under various abiotic stress conditions at different stages of development in rice. We established the nuclear localization of OsHOX22 and OsHOX24 proteins, analyzed their transactivation and dimerization properties, and identified their novel interacting proteins. Further, we studied the binding property of purified proteins with specific DNA sequences and identified their putative downstream targets at whole genome-level. In addition, we over-expressed *OsHOX24* in *Arabidopsis* and showed its role in abiotic stress responses.

## Materials and Methods

### Plant Growth Conditions, Stress, and Hormone Treatments

Wild-type *Arabidopsis* (Col-0) seeds procured from *Arabidopsis* Biological Research Centre (ABRC) were grown in pots containing autoclaved mixture of agropeat and vermiculite (1:1) [supplemented with nutrient medium (Somerville and Ogren, 1982)] or on MS plates as described previously (Laxmi et al., 2004; Jain et al., 2006b; Sharma et al., 2014; Maloney et al., 2015). The seedlings were grown in a culture room with ∼100 μmol m^-2^ s^-1^ light maintained at 20 ± 1°C with 14/10 h of day/night photoperiod. Expression profiling of candidate homeobox genes in different tissues/organs/developmental stages of rice (*Oryza sativa* ssp. *indica* IR64 cultivar) and after treatment with various hormones [50 μM indole-3-acetic acid (IAA), 10 μM epibrassinolide (EBR), 100 μM ABA, 100 μM salicylic acid (SA), 50 μM 1-aminocyclopropane-1-carboxylic acid (ACC), 50 μM benzyl aminopurine (BAP) and 50 μM gibberellic acid (GA3)] was performed as described previously (Jain et al., 2006a,b, 2008; Wang et al., 2009 ; Yue et al., 2015). Relatively higher concentrations of different hormones were used, since treatments were given to whole rice seedlings hydroponically for short duration (3 h) to study differential gene expression.

For imparting abiotic stresses, 7-day-old rice seedlings were removed from trays and subjected to desiccation (whole seedlings were kept between folds of tissue paper and allowed to dry), salinity (seedling roots were submerged in 200 mM NaCl solution), cold [seedling roots were submerged in reverse-osmosis (RO) water and kept at 4 ± 1°C in cold room] and osmotic stress (seedling roots were submerged in 200 mM mannitol solution) treatments as described earlier (Jain et al., 2008). Whole seedlings under control and stress conditions were harvested after 1, 3, 6, and 12 h time points and snap frozen in liquid nitrogen. Likewise, greenhouse grown 5-week-old mature rice plants were subjected to desiccation and salinity stress treatments for 1, 3, 6, and 12 h followed by tissue harvesting. Seedlings kept in RO water and plants grown in pots (filled with soil) supplied with RO water served as experimental control. Four-month-old reproductive-stage rice plants were subjected to desiccation (by withholding water) and salinity (200 mM NaCl solution) stresses, and flag-leaf and panicle tissues were harvested after 6 and 12 h.

### Real-Time Polymerase Chain Reaction (PCR) Analysis

To study the gene expression, quantitative real-time PCR analysis was carried out as described earlier (Sharma et al., 2014). At least two biological replicates for each sample and three technical replicates for each biological replicate were analyzed. The relative expression level of each gene was determined using ΔΔ*C*_T_ calculation as described previously (Jain et al., 2006b). To normalize the relative mRNA level of individual genes in different RNA samples, *PP2A* and *UBQ5* were used as most suitable internal control genes for *Arabidopsis* and rice, respectively (Sharma et al., 2014). The list of primers used for real-time PCR is given in **Supplementary Table S1**.

### Sequence Analysis and Homology Modeling

The alignment of genomic and coding sequences of homeobox genes was done using Sim4 software (Florea et al., 1998) to determine exon–intron organization. For promoter analysis, 2 kb sequence from upstream of start codon of the homeobox genes was retrieved using corresponding BAC/PAC clone sequences from the National Centre for Biotechnology Information (NCBI). The sequences were used as query in the PLACE database^1^ to identify *cis*-regulatory stress-responsive elements.

The homology modeling for HD of OsHOX22 and OsHOX24 proteins was performed using 9ANT (Antennapedia homeodomain–DNA complex) from *Drosophila* (Fraenkel and Pabo, 1998) under default parameters in Modeller (version 9.11) and visualized by PyMol software (version 1.6). The modeled structures were assessed by Ramachandran plot analysis in RAMPAGE^2^.

### Subcellular Localization of Recombinant Homeobox Proteins

The full-length and truncated versions [C-terminal deletion (ΔC) of 165-261 amino acid (aa) for OsHOX24ΔC and 174-276 aa for OsHOX22ΔC] of homeobox genes were PCR amplified using gene-specific primers (**Supplementary Table S2**) and cloned in psGFPcs vector (Kapoor et al., 2002) using *Apa*I and *Xma*I restriction sites. The N-terminal GFP fusion constructs and empty vector (psGFPcs; experimental control) were transiently transformed in onion epidermal cells via particle bombardment method using PDS-1000 He particle delivery system (Bio-Rad Laboratories, Hercules, CA, USA) as described earlier (Sharma et al., 2014). The transformed cells were incubated in dark at 23°C for 24 h and onion peels were visualized under confocal microscope (AOBS TCS-SP2, Leica Microsystems, Mannheim) for detection of GFP and DAPI signals.

### Transactivation and Dimerization Assays

Full-length coding sequences of *OsHOX24* (786 bp), *OsHOX22* (831 bp), and their C-terminal deleted regions, *OsHOX24ΔC* (495 bp) and *OsHOX22ΔC* (521 bp), were PCR amplified using gene-specific primers (**Supplementary Table S2**) and cloned into pGBKT7 vector containing GAL4 DNA-binding domain. The confirmed constructs were transformed in yeast strain (AH109) [harboring *HIS3*, *ADE2*, *MEL1*, and *lacZ* reporter genes] according to small-scale yeast transformation procedure (Clontech). The empty vector (pGBKT7) and pGBKT7-p53 + pGADT7-T antigen transformed in yeast served as negative and positive experimental controls, respectively. The transformants were further serially diluted and dropped on various SD selection media, namely SD-Trp, SD-Trp-His, and SD-Trp-His-Ade and incubated at 30°C for 3–5 days. To check the dimerization properties of homeobox proteins, full-length coding sequence of *OsHOX24*, *OsHOX22*, and their C-terminal deleted regions, *OsHOX24ΔC* and *OsHOX22ΔC*, were cloned into pGADT7 vector containing GAL4 activation domain. The bait vector containing truncated version of ORFs were co-transformed with prey vector containing either full-length or truncated ORFs in yeast. The empty vectors, pGBKT7 + pGADT7, and pGBKT7-p53 + pGADT7-T antigen, cotransformed in yeast were used as negative and positive controls, respectively. The transformants were grown on SD-Trp-Leu and SD-Trp-Leu-His-Ade selection media and incubated at 30°C for 3–5 days.

### Yeast-two Hybrid Analysis

The C-terminus deletion constructs, OsHOX24ΔC (1–164 aa) and OsHOX22ΔC (1–173 aa) were created in bait vector for yeast-two hybrid analysis. A cDNA library was generated from 3 h drought stress treated 7-day-old rice seedlings in library vector, pGADT7-Rec, by recombination-based cloning using *Sma*I and transformed in yeast (AH109), according to manufacturer’s instructions (Clontech). Using large-scale transformation by PEG/LiAc method, bait constructs (OsHOX24ΔC and OsHOX22ΔC) were transformed in the competent cells prepared from a single aliquot of cDNA library glycerol stock, according to instructions provided by manufacturer (Clontech). The transformation mixture was spread on SD selection media, SD-Trp-Leu-His and incubated at 30°C for 5 days until colonies appeared. Selected transformed yeast colonies were streaked on SD-Trp-Leu media, and simultaneously screened by colony PCR using AD5′ and AD3′ library vector-specific primers to check the presence of inserts. The colonies possessing insert size > 300 bp were streaked on SD-Trp-Leu-His (triple dropout medium; TDO medium) and SD-Trp-Leu-His-Ade (quadruple dropout medium; QDO medium) for reconfirmation. The putative clones were streaked on TDO and QDO selection medium supplemented with either 40 mg/ml X-α-Gal or X-β-Gal also, and allowed to grow at 30°C for 3–5 days till blue color development. On the basis of color development in colonies due to activation of *Mel1*/*lacZ* reporter gene and size of insert, selected clones were confirmed by Sanger sequencing.

Further, the interacting partners of homeobox TFs were confirmed by drop tests and *lacZ* reporter gene quantitative assay using *O*-nitrophenyl-beta-D-galactopyranosidase (ONPG) as substrate, according to manufacturer’s instructions (Clontech). pGBKT7-Lam + pGADT7-T, and pGBKT7-p53 + pGADT7-T antigen, were used as negative and positive experimental control, respectively. The β-galactosidase unit for each sample was calculated according to Miller (1972). The experiments were performed in three biological replicates. To study the expression profiles of homeobox genes and the genes encoding for their interacting proteins under abiotic stress conditions, we analyzed the publicly available microarray data from Genevestigator v.3^3^. The heatmaps depicting log_2_ ratio (fold change) values of the respective genes during drought, salinity and cold stress conditions were generated.

### Electrophoretic Mobility Shift Assay (EMSA)

The PCR amplified complete ORFs [using gene-specific primers (**Supplementary Table S2**)] of *OsHOX24* and *OsHOX22* were cloned in pET28a expression vector, in *Bam*HI/*Eco*RI and *Xho*I/*Hin*dIII restriction sites, respectively. Recombinant protein induction followed by purification under native conditions was carried out as described earlier (Sharma et al., 2014). For EMSA, single-stranded biotinylated and HEX-labeled AH1 and AH2 oligonucleotide sequences, synthesized commercially (Sigma) as tetrameric repeats [oligos with four consecutive repeats of *cis*-regulatory motifs (AH1/AH2)], were annealed in equimolar volumes. For protein–DNA binding reactions, 25–50 nM annealed oligos (HEX or biotin-labeled AH1/AH2) were added to 5–10 μg of purified proteins along with 5X DNA binding buffer (50 mM Tris-Cl pH-8.0, 2.5 mM EDTA, 2.5 mM DTT, 5 mM MgCl_2_, 5X protease inhibitor, 250 mM KCl, and 12.5% glycerol). Reactions devoid of annealed oligos or purified proteins served as experimental control. For binding experiments performed with HEX-labeled oligos, 200-fold excess of unlabelled oligos and (1 μg/μl) poly(di-DC) were used as specific and non-specific competitor DNA, respectively. The binding reactions were incubated at room temperature for 30 min followed by 6% native polyacrylamide gel electrophoresis in 0.25X Tris-borate-EDTA (TBE) at 15–20 mA for 30 min. HEX-labeled fluorescent oligos complexed with purified proteins were visualized directly under Typhoon scanner. For biotin-labeled oligos complexed with purified proteins, nylon membrane (+vely charged) was used for electrophoretic transfer followed by UV crosslinking and incubation with streptavidin-horseradish peroxidase conjugate/blocking reagent solution (1:300 dilution) for requisite time. The protein–DNA complexes were detected via chemiluminescence using Enhanced Chemiluminescence (ECL) Detection system (GE Healthcare, Buckinghamshire, UK) as per manufacturer’s instructions.

### Over-expression of *OsHOX24* in *Arabidopsis*

To over-express *OsHOX24* in *Arabidopsis*, the PCR amplified complete ORF [using gene specific primers (**Supplementary Table S2**)], was cloned in binary expression vector, pBI121, in *Xba*I*/Bam*HI restriction sites. The confirmed clone was transformed in *Agrobacterium* strain GV3101 for generating *Arabidopsis* transgenic lines. The transformation of WT (Col-0) *Arabidopsis* plants was done using *Agrobacterium* strain harboring the confirmed construct via floral-dip method (Clough and Bent, 1998). Seeds obtained from transformed *Arabidopsis* plants were screened on MS medium supplemented with kanamycin. The PCR-positive transgenic lines were grown till homozygous stage for future analyses as described earlier (Jain et al., 2006b).

### Phenotypic and Stress Assays

To study the effect of *OsHOX24* transgene in *Arabidopsis*, phenotype of over-expression transgenic lines was compared with WT at different stages of plant development. To study the response of *Arabidopsis* transgenics under various abiotic stress conditions, seed germination assays were carried out as described earlier (Sharma et al., 2014). WT *Arabidopsis* and transgenic seeds were plated on MS medium without or with ABA (0.5, 1, and 5 μM) or NaCl (100, 200, and 300 mM), subjected to stratification (at 4°C) in dark for 2 days and seed germination [radical emergence after rupture of seed testa (Jain et al., 2006b; De Giorgi et al., 2015)] was recorded after 3 days of transfer to light. To assess the effect of desiccation stress, WT and 35S::*OsHOX24* transgenics (HZIP1-2.3 and HZIP1-8.2) were grown on MS medium supplemented without or with PEG6000 (-0.4 MPa) for 10 days. The root length and fresh weight of seedlings grown under control and desiccation stress conditions were measured. The relative average root length and fresh weight under desiccation stress condition were calculated as percentage of root length and fresh weight of seedlings under control condition.

Four-week-old mature plants were subjected to desiccation stress by withholding water for 3 weeks followed by 1-week recovery. WT and transgenic plants of same age served as experimental controls. Plant growth was monitored till seed maturation and phenotypes under control and desiccation stress followed by recovery phase were documented. To assess the effect of desiccation stress, chlorophyll content of leaves of transgenic and WT plants under desiccation and control conditions was estimated as described earlier (Sharma et al., 2014).

### Microarray Analysis

Total RNA was isolated from 10-day-old *Arabidopsis* seedlings (WT and 35S::*OsHOX24* transgenics) and quality control was performed as described earlier (Sharma et al., 2014). Microarray analysis for three independent biological replicates was conducted using Affymetrix GeneChip 3′ IVT kit (Affymetrix, Santa Clara, CA, USA) according to manufacturer’s instructions, as described earlier (Sharma et al., 2014). Microarray data has been submitted in the Gene Expression Omnibus database at NCBI under the series accession number GSE79188. GO enrichment was carried out using online GOEAST toolkit. The metabolic pathway analysis was carried out in AraCyc database as described previously (Sharma et al., 2014). Heatmaps were generated using MeV (version 4.9). Validation of microarray experiment for selected differentially expressed genes was carried out by real-time PCR analysis using gene-specific primers (**Supplementary Table S1**).

### Statistical Analysis

All the experiments were conducted in at least three biological replicates unless otherwise mentioned and SE was computed in each case. For the estimation of statistical significance, Student’s *t-*test was performed. The data points representing statistically significant differences between WT and transgenic lines or between control and stress conditions have been indicated.

## Results

### Sequence Analysis, Domain Organization, and DNA Binding

Two of the homeobox genes belonging to HD-ZIP I subfamily, *OsHOX22* and *OsHOX24*, which showed up-regulation under abiotic stresses in our previous study (Jain et al., 2008), were selected for further characterization and functional validation in this study. For *OsHOX24*, cDNA clone (AK063685) was obtained from National Institute of Agrobiological Sciences (NIAS). However, we observed ambiguity in the annotated sequence of *OsHOX22* at Rice Genome Annotation Project (RGAP) and corresponding cDNA clone (AK109177) sequence. The ORF length of *OsHOX22* in RGAP corresponded to 831 bp in contrast to NIAS cDNA clone, which corresponded to ORF length of 570 bp. Therefore, we amplified *OsHOX22* cDNA via reverse transcriptase-PCR (RT-PCR; from total RNA isolated from 3 h drought stress treated 7-day-old rice seedlings) and cloned in pGEMT-Easy vector. The sequencing results confirmed the annotated sequence reported in RGAP (LOC_Os04g45810).

The gene sequences of *OsHOX24* and *OsHOX22* were found to be of 1423 and 1347 bp lengths, respectively, harboring two exons interrupted by a single intron (phase 0) each (**Supplementary Figure S1A**). The ORFs of *OsHOX24* and *OsHOX22* comprised of 786 and 831 bp encoding 261 and 276 aa residues, respectively. The domain organization of OsHOX24 and OsHOX22 proteins revealed the presence of highly conserved HD and HALZ domains (**Supplementary Figure S1A**). A putative monopartite NLS was also detected within the HD region of both the homeobox proteins (**Supplementary Figure S1A**).

We identified several *cis*-regulatory elements in the promoter sequences (2 kb upstream) of *OsHOX24* and *OsHOX22* (**Supplementary Figure S2**). Many of these *cis*-regulatory motifs were found to be stress-responsive in nature, for example, ABA-responsive element (ABRE), C-repeat binding factor-dehydration responsive element (CBF-DRE), low temperature response element (LTRE), myeloblastosis element (MYB), MYB core element (MYBCORE), and myelocytomatosis element (MYC). These *cis*-regulatory elements have been reported to be vital for the regulation of stress-responsive genes in plants (Liu et al., 2014).

The availability of crystal structure of *Drosophila* Antennapedia HD protein–DNA complex (Protein Data Bank code 9ANT; Fraenkel and Pabo, 1998) enabled us to determine the three-dimensional structure of the HD of homeobox proteins by homology modeling (**Supplementary Figure S3**). The HD portions of OsHOX24 and OsHOX22 homeobox proteins exhibited 41–43% identity and showed more than 85% coverage of the template structure. The modeled HD structures of OsHOX24 and OsHOX22 were found to possess three alpha helices interconnected by loops (**Supplementary Figure S3**). By comparing the modeled HD structures of OsHOX24 and OsHOX22 with template, the residues forming nucleotide-binding site were identified (**Supplementary Figures S3A,C**). It was observed that HD of both the homeobox proteins were capable of binding with DNA on the major groove by forming hydrogen bonding via three amino acid residues, namely Arg4, Ile46, and Asn49 (**Supplementary Figures S3B,D**), conserved between the model and template. Ramachandran plot analysis showed the presence of 98 and 100% of the residues in the modeled HD structures of OsHOX24 and OsHOX22, respectively, lie in the favored regions.

### Homeobox Genes Were Highly Induced during Abiotic Stress Conditions at Different Stages of Development

**FIGURE 1:**
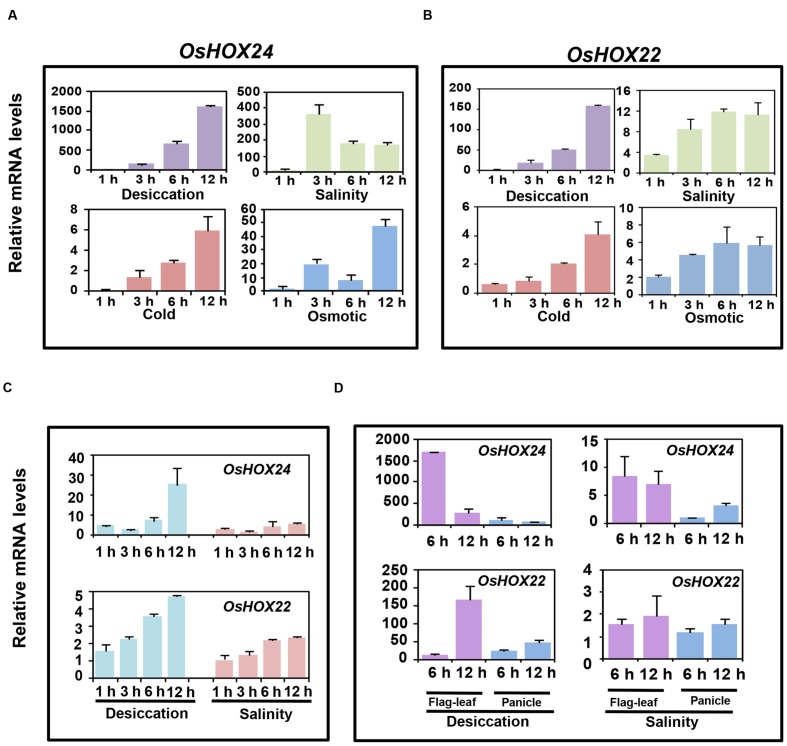
**Differential expression of homeobox genes under different abiotic stress conditions at various stages of development.** **(A,B)** Real-time PCR analysis of *OsHOX24* **(A)** and *OsHOX22* **(B)** using the total RNA isolated from 7-day-old seedlings subjected to desiccation, salinity, cold, and osmotic stresses. **(C)** Real-time PCR analysis of mature plants (5-week-old) subjected to desiccation and salinity stresses for 1, 3, 6, and 12 h time points. **(D)** Real-time PCR analysis of the total RNA isolated from flag-leaf and panicle of reproductive stage (4-month-old) plants which were subjected to desiccation and salinity stresses for 6 and 12 h time points. The mRNA levels of *OsHOX24* and *OsHOX22* genes under different stresses were calculated relative to the gene expression in control seedlings/tissues. Values are mean (*N* = 3) from three representative biological replicates. Error bars indicate SE.

We confirmed the differential expression of *OsHOX24* and *OsHOX22* via real-time PCR analysis in various developmental stages of rice (**Supplementary Figures S1B,C**); as reported previously (Jain et al., 2008). Further, we performed comprehensive expression profiling of these genes under abiotic stress conditions at different stages of rice development. *OsHOX24* and *OsHOX22* genes were highly up-regulated in rice seedlings subjected to desiccation, salinity, cold, and osmotic stress treatments for various durations (1, 3, 6, and 12 h), as revealed by real-time PCR analysis (**Figures 1A,B**). The transcript levels of *OsHOX24* and *OsHOX22* gradually increased with the duration of stress treatment in all the cases. The up-regulation of *OsHOX24* and *OsHOX22* was higher in the seedlings subjected to desiccation stress as compared to other stresses (**Figures 1A,B**). Notably, the transcript level of *OsHOX24* was much more elevated than *OsHOX22* under different stress conditions except cold stress (**Figures 1A,B**). For instance, after 12 h of desiccation stress, the accumulation of *OsHOX24* transcripts was about 10 times more than *OsHOX22* in the rice seedlings (**Figures 1A,B**).

Further, the transcript level of *OsHOX24* and *OsHOX22* was analyzed in 5-week-old mature plants, subjected to desiccation and salinity stresses for 1, 3, 6, and 12 h. Significant up-regulation of *OsHOX24* and *OsHOX22* was detected in the mature rice plants on exposure to stress and prolonged exposure led to further increase in their transcript levels (**Figure 1C**). The extent of up-regulation of homeobox genes due to desiccation stress was found to be slightly more as compared to salinity stress. Both the homeobox genes showed increase in transcript levels till 6 h under desiccation and salinity stresses. After extended period of desiccation stress (12 h), the transcript level of *OsHOX24* was found to be about five times more than *OsHOX22* (**Figure 1C**).

Next, we examined the expression profiles of homeobox genes in panicle and flag-leaf of 4-month-old (reproductive stage) rice plants, subjected to mock, desiccation and salinity stresses for 6 and 12 h. The analysis revealed up-regulation of *OsHOX24* and *OsHOX22* in both flag-leaf and panicle during desiccation stress (**Figure 1D**). It was also observed that the extent of up-regulation was more in flag-leaf than panicle during desiccation stress. The transcript levels of *OsHOX24* were induced in flag-leaf within 6 h of desiccation stress (**Figure 1D**). However, the enhanced transcript levels of *OsHOX22* were detected in flag-leaf only after 12 h of desiccation stress (**Figure 1D**). In case of salinity stress, the transcript level of *OsHOX24* was found to be up-regulated in both flag-leaf and panicle tissues (**Figure 1D**). However, even after 12 h of salinity stress, no significant up-regulation of *OsHOX22* could be detected in either of the tissues analyzed (**Figure 1D**).

### Differential Expression of Homeobox Genes in Response to Plant Hormones

**FIGURE 2:**
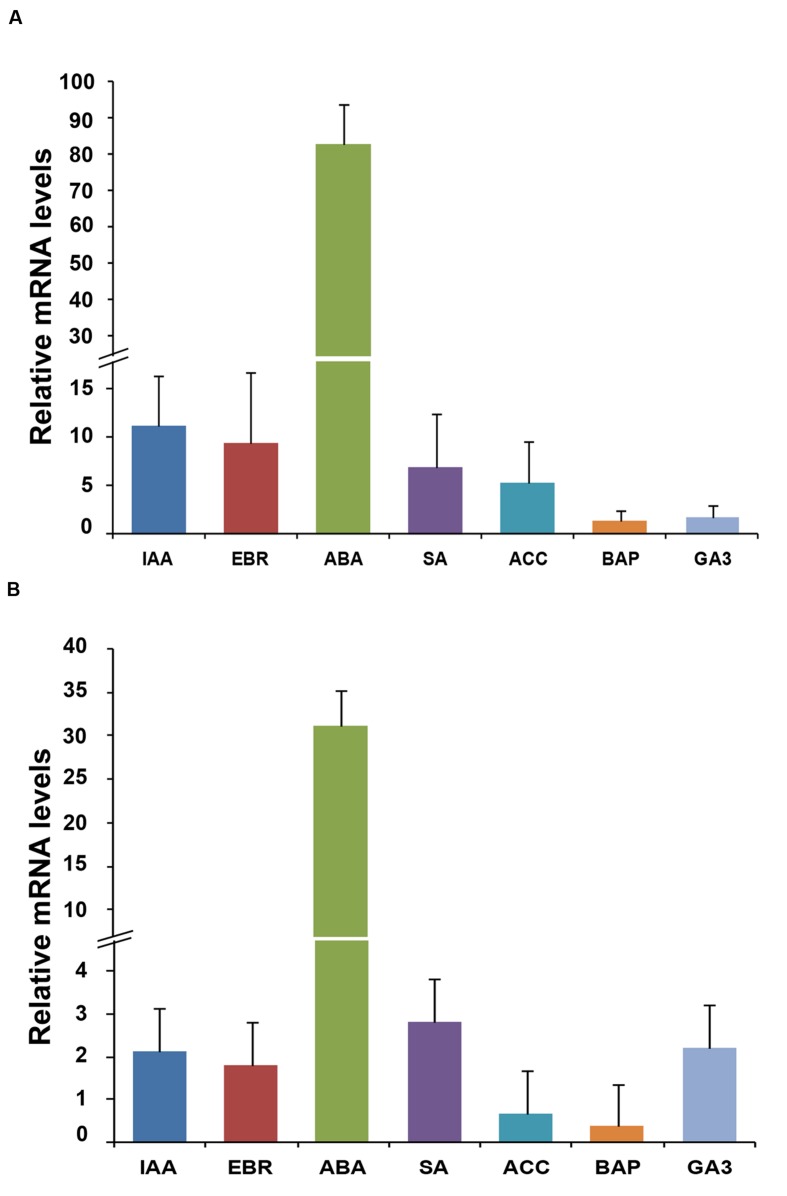
**Differential expression of homeobox genes during hormone treatments.** **(A,B)** Real-time PCR analysis of *OsHOX24* **(A)** and *OsHOX22* **(B)** using total RNA isolated from 7-day-old seedlings subjected to various hormone treatments for 3 h. The mRNA levels were calculated relative to the expression in mock-treated control seedlings (experimental control). IAA, (50 μM indole-3-acetic acid); EBR, (10 μM epibrassinolide); ABA, (100 μM abscisic acid); SA, (100 μM salicylic acid); ACC, (50 μM 1-aminocyclopropane-1-carboxylic acid); BAP, (50 μM benzyl aminopurine) and GA3 (50 μM gibberellic acid). Values are mean (*N* = 3) from three representative biological replicates. Error bars indicate SE.

To study the effect of plant hormones, the transcript profiling of homeobox genes was carried out in the rice seedlings subjected to various hormone treatments exogenously, including IAA, EBR, ABA, SA, ACC, BAP, and GA3. The transcript levels of *OsHOX24* and *OsHOX22* genes were found to be elevated under different hormone treatments (**Figure 2**). ABA treatment resulted in significant increase (30–80-fold) in the transcript levels of both *OsHOX24* and *OsHOX22*. The transcript level of *OsHOX24* was induced in the presence of IAA, EBR, SA, and ACC as well (**Figure 2A**), whereas, highest up-regulation of *OsHOX22* was found in the presence of SA followed by GA3 and IAA (**Figure 2B**). These results suggested that homeobox genes are involved in ABA or other hormone-signaling pathways in rice.

### Recombinant Homeobox Proteins Are Nuclear-Localized

**FIGURE 3:**
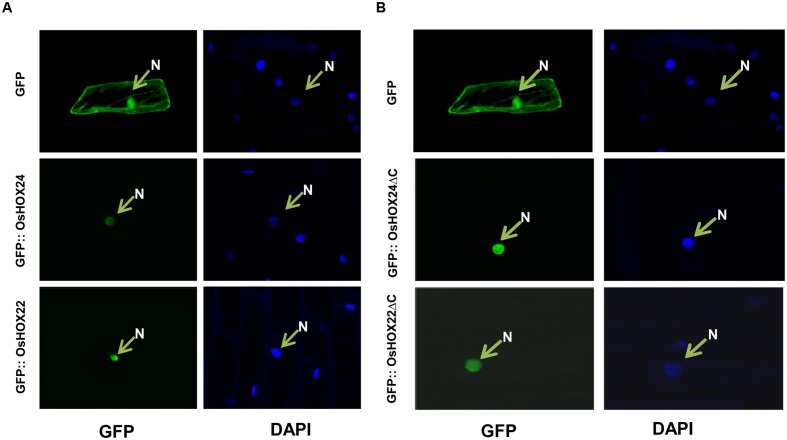
**Sub-cellular localization of full-length and truncated (ΔC) OsHOX proteins.** **(A,B)** Sub-cellular localization of full-length (GFP::OsHOX24 and GFP::OsHOX22) **(A)** and truncated (GFP::OsHOX24ΔC and GFP::OsHOX22ΔC) **(B)** fusion proteins in onion epidermal cells. Empty GFP vector was used as experimental control. The left panel shows GFP fluorescence followed by DAPI (nucleus (N)-specific dye) staining in the right panel for each construct and vector control.

The amino acid sequence analysis of OsHOX24 and OsHOX22 proteins revealed the presence of a putative monopartite NLS within their HD. To confirm the subcellular localization, their complete ORFs were cloned in psGFPcs vector with N-terminal GFP fusion. The GFP-fused full-length homeobox proteins (GFP::OsHOX24 and GFP:: OsHOX22) were transiently expressed in onion epidermal cells. In case of empty vector (GFP alone), fluorescence was spread throughout the onion cell, whereas for full-length recombinant homeobox proteins, fluorescence was detected only in the nucleus, indicating the nuclear-localization of homeobox proteins (**Figure 3A**). Further, we deleted the C-terminal transactivation domain of homeobox proteins (165–261 aa for OsHOX24ΔC and 174–276 aa for OsHOX22ΔC) and performed subcellular localization studies in onion epidermal cells. The truncated recombinant proteins were also found to be localized in the nucleus (**Figure 3B**). The nuclear-localization of recombinant homeobox proteins was further confirmed by staining with nucleus-specific dye, DAPI.

### DNA Binding of Homeobox Proteins and Identification of Putative Targets

**FIGURE 4:**
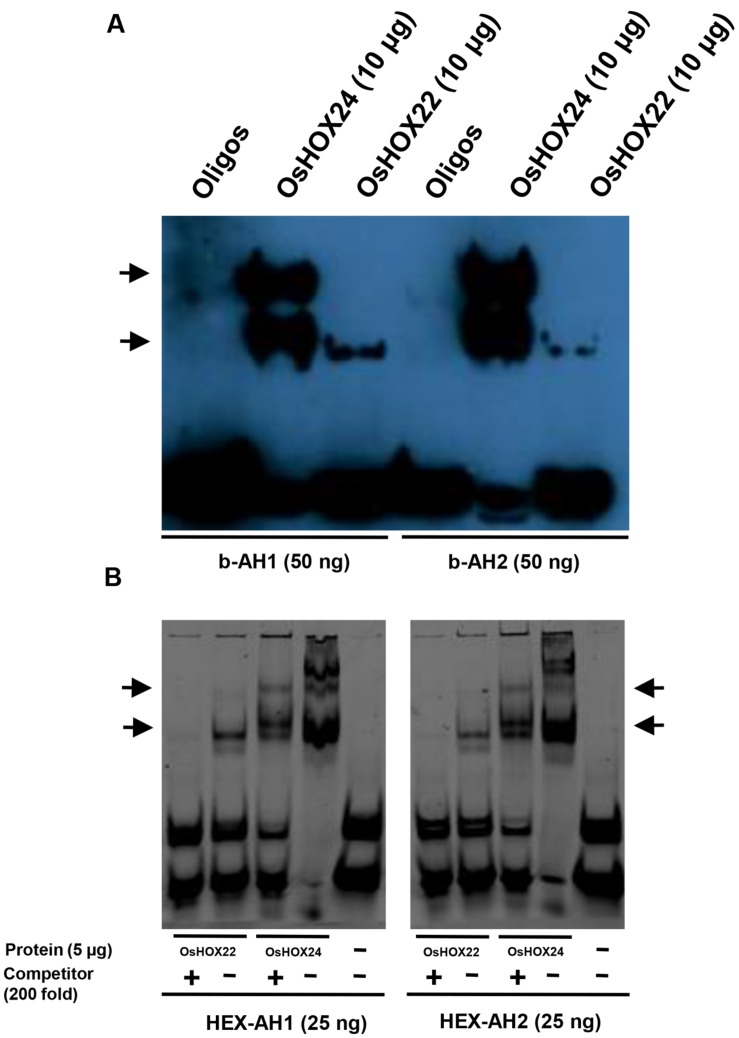
**Binding of homeobox TFs to specific DNA (AH1 and AH2) motifs.** **(A,B)** EMSA showing binding of biotinylated AH1 (b-AH1) and AH2 (b-AH2) tetrameric motifs with purified recombinant proteins, 6xHis::OsHOX24 and 6xHis::OsHOX22 **(A)** and binding of HEX-labeled AH1 and AH2 tetrameric motifs with purified recombinant proteins, 6xHis::OsHOX24 and 6xHis::OsHOX22 **(B)**. For binding reactions, 25–50 nM of annealed oligos were used along with 5–10 μg purified protein and samples were run on 6% native PAGE in 0.25X TBE buffer, followed by development of blot (biotinylated oligos) or direct visualization (HEX-labeled oligos). The arrows indicate the position of binding of recombinant protein with tetrameric motifs as detected by streptavidin-HRP conjugate. For EMSA experiment using HEX-labeled oligos, 200-fold excess of unlabelled oligos were used as competitor in a separate reaction.

Earlier studies have demonstrated the specific binding of HD-ZIP I class members with 9 bp pseudopalindromic sequences, namely AH1 (CAAT(A/T)ATTG) and AH2 (CAAT(C/G)ATTG; Sessa et al., 1997; Palena et al., 1999; Meijer et al., 2000). We also studied the binding of purified homeobox proteins with tetrameric oligos, AH1 and AH2, via EMSA. OsHOX24 and OsHOX22 proteins were found to bind with biotinylated AH1 and AH2 tetrameric oligos (**Figure 4A**). The presence of multiple bands indicated that OsHOX24 could possibly associate with tetrameric oligos in monomeric or oligomeric forms. Similar patterns of protein–DNA binding could be detected using HEX-labeled oligos as well. Incorporation of 200-fold molar excess of unlabelled oligos as competitor abolished the DNA–protein binding for OsHOX22, whereas highly reduced concentration of DNA–protein complex was observed for OsHOX24 (**Figure 4B**). These results indicate that OsHOX24 possesses stronger binding affinity for these target motifs as compared to OsHOX22.

The genes harboring AH1 and/or AH2 motifs in their promoters may represent the downstream targets of homeobox proteins. Therefore, we scanned 1 kb upstream regions of all rice protein coding genes (39,045) for the presence of AH1 and/or AH2 motifs. At least 809 rice genes possessing one or more of these target motifs in their promoter regions were identified. A larger number (539 genes) of rice genes harbored AH1 motif as compared to the AH2 motif (289 genes; **Supplementary Table S3**). We investigated the major functional categories represented among these genes via GO enrichment analysis. In biological process category, the genes involved in small molecule metabolic processes, lipid metabolic process, cellular response to stimulus, oxidation-reduction, hormone mediated signaling pathways and reproductive and anatomical structure developmental processes were found to be significantly enriched (**Supplementary Figure S4**).

### Homeobox Proteins Display Transactivation and Dimerization Properties

**FIGURE 5:**
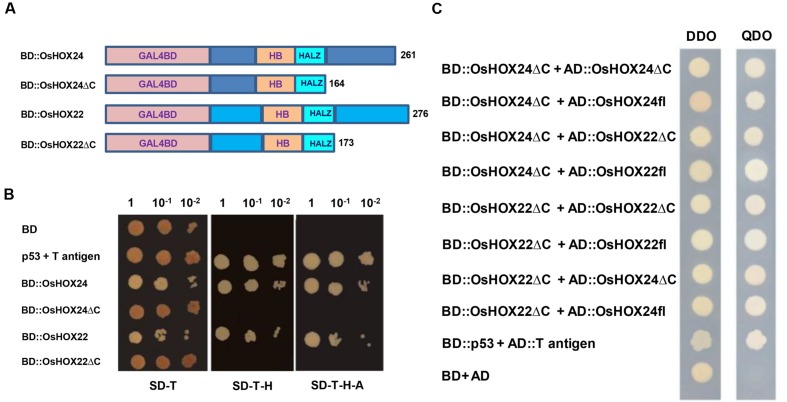
**Transactivation and dimerization properties of homeobox proteins.** **(A)** Schematic representation of full-length homeobox proteins (BD::OsHOX24 and BD::OsHOX22) and truncated (C-terminal acidic region removed) fusion constructs (BD::OsHOX24ΔC and BD::OsHOX22ΔC) used. **(B)** Transactivation assay of full-length and truncated (ΔC) homeobox proteins in yeast. The transformants grown on SD-Trp (SD-T, left) medium; SD-Trp-His medium (SD-T-H, middle) and SD-Trp-His-Ade (SD-T-H-A, right) medium are shown. **(C)** Dimerization assay of full-length (fl) and truncated (ΔC) homeobox proteins. The deletion constructs, BD::OsHOX24ΔC and BD::OsHOX22ΔC, were co-transformed with different combinations of full-length (AD::OsHOX24fl, AD::OsHOX22fl) and deletion constructs (AD::OsHOX24ΔC, AD::OsHOX22ΔC) of homeobox proteins in yeast, as indicated on left side panel. The transformants were grown on SD-Trp-Leu (DDO medium) and SD-Trp-Leu-His-Ade medium (QDO medium) for confirmation of interaction. pGBKT7-p53 + pGADT7-T antigen represents positive control. Empty pGBKT7 vector (BD) represents negative control for transactivation assay and pGBKT7 + pGADT7 represents negative control for dimerization assay.

OsHOX24 and OsHOX22 proteins were found to be rich in acidic amino acids at the C-terminal region, which could possibly contribute to their transactivation property. Thus, we investigated the transcriptional activation property of these HD-ZIP I TFs in yeast. The complete ORFs and C-terminal deletion constructs (ΔC) of *OsHOX24* and *OsHOX22* were cloned in yeast expression vector containing DNA binding domain (**Figure 5A**). The colonies of transformed yeast cells grew uniformly on SD-Trp selection medium. The growth of yeast transformants on SD-Trp-His and SD-Trp-His-Ade selection media, even with increasing serial dilution, confirmed the transactivating nature of full-length homeobox proteins (**Figure 5B**). In contrast, yeast transformants harboring *OsHOX24*Δ*C* and *OsHOX22*Δ*C*, and empty bait vector control, did not grow in either of the selection media. This suggested that C-terminal region of full-length homeobox proteins was responsible for their transcriptional activation property, because these proteins could drive the expression of *HIS3* and *ADE2* reporter genes even in the absence of any interacting protein in yeast.

Various homeobox proteins belonging to HD-ZIP class have been reported to form homodimers or heterodimers with other members (Meijer et al., 2000). This prompted us to investigate about the dimerization property of OsHOX24 and OsHOX22 in yeast. The complete ORFs of *OsHOX24* and *OsHOX22* and their C-terminal deletion constructs (*OsHOX24*Δ*C* and *OsHOX22*Δ*C*) were cloned in pGADT7 vector. The bait vector containing truncated version of homeobox proteins was co-transformed with prey vector harboring either full-length or truncated homeobox proteins in yeast. Several colonies were obtained on SD-Trp-Leu-His-Ade selection media for all the combinations of cotransformed bait and prey plasmid constructs, except for the negative control, indicating that the full-length and truncated versions of homeobox proteins can homodimerize and heterodimerize with each other (**Figure 5C**). These observations suggest that C-terminal region of these homeobox proteins may not be important for dimerization.

### Identification of Novel Interacting Proteins of Homeobox Proteins and their Gene Expression Profiling

**FIGURE 6:**
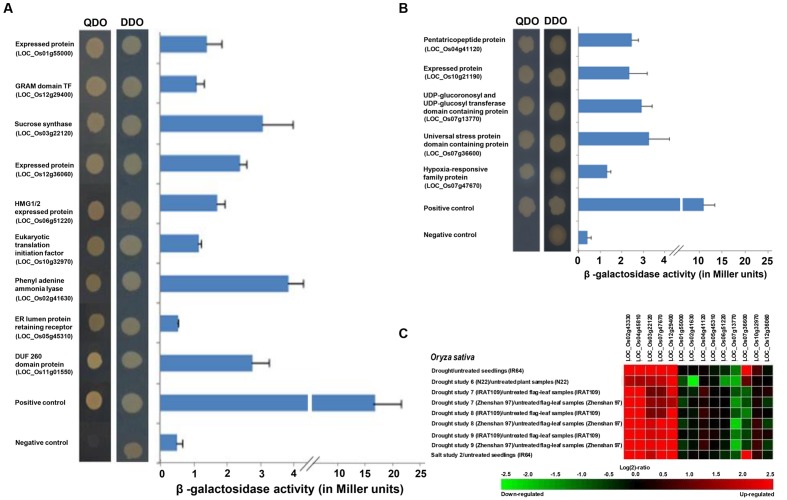
**Interacting proteins of homeobox TFs identified by yeast-two hybrid analysis and their gene expression profiling.** **(A,B)** The transformants in yeast strain were grown on SD-Trp-Leu (DDO medium) and SD-Trp-Leu-His-Ade medium (QDO medium) for confirmation of interaction of proteins with OsHOX24 **(A)** and OsHOX22 **(B)**. pGBKT7-p53 + pGADT7-T antigen represents positive control and pGBKT7-Lam + pGADT7-T antigen represents negative control. The graphical panels (right) represent quantitative β-galactosidase assay showing the *lacZ* reporter gene expression (β-galactosidase activity in Miller units) for interacting proteins of OsHOX24 **(A)** and OsHOX22 **(B)**. Ortho-nitrophenyl-β-D-galactoside (ONPG) was used as substrate for β-galactosidase assay. The putative function and locus identifier of the interacting proteins are given on the left side. **(C)** Heat-map showing gene expression profiles of *OsHOX24*, *OsHOX22*, and genes encoding for their interacting proteins during various abiotic stress conditions. The heat-map has been generated by Genevestigator (v.3) using the publicly available abiotic stress related microarray data. The color scale representing fold change (log_2_ ratio) is shown below the heat-map.

The deletion constructs of homeobox genes (*OsHOX24*Δ*C* and *OsHOX22*Δ*C*) were used as baits to identify their interacting proteins. Numerous transformants were obtained after large-scale transformation of *OsHOX24*Δ*C* and *OsHOX22*Δ*C* bait plasmid DNAs and screened on SD media lacking leucine, tryptophan and histidine. Selected transformants were screened by colony PCR and further grown on TDO (SD-Trp-Leu-His) and QDO (SD-Trp-Leu-His-Ade) media supplemented with or without X-α-Gal or X-β-Gal for reconfirmation. The growth of putative clones and blue color development in colonies was observed on TDO and QDO selection media, which also indicated activation of reported genes (*Mel1* and *lacZ*). The sequencing of plasmid DNAs of selected confirmed clones resulted in the identification of interacting proteins of candidate homeobox TFs. At least nine and five proteins were identified as interacting proteins of OsHOX24 and OsHOX22, respectively. OsHOX24 was found to interact with protein fragments belonging to GRAM domain TF, expressed protein, high mobility group protein (HMG1/2), eukaryotic translation initiation factor I, DUF domain protein, endoplasmic reticulum (ER) lumen protein retaining receptor and enzymes like sucrose synthase and phenylalanine ammonia lyase (**Figure 6A**). OsHOX22 was found to interact with protein fragments belonging to an expressed protein, pentatricopeptide repeat protein, hypoxia-responsive family protein, universal stress protein domain containing protein and UDP-glucuronosyl and UDP-glucosyl transferase domain containing protein (**Figure 6B**).

Further, the interacting proteins were examined for the activation of *lacZ* reporter gene via ONPG assay. We observed a considerable difference in the β-galactosidase activity of putative interacting proteins. Among the OsHOX24 interactors, phenylalanine ammonia lyase showed highest β-galactosidase activity followed by sucrose synthase, DUF domain protein, expressed protein, HMG1/2 expressed protein, GRAM domain TF and eukaryotic translation initiation factor I, whereas least β-galactosidase activity (almost comparable with negative control) was shown by ER lumen protein retaining receptor (**Figure 6A**). In case of OsHOX22 interactors, universal stress protein domain containing protein showed highest β-galactosidase activity followed by UDP-glucuronosyl and UDP-glucosyl transferase domain containing protein, pentatricopeptide protein, expressed protein and hypoxia-responsive family protein (**Figure 6B**).

The expression profiles of the genes encoding for interacting proteins of OsHOX24 and OsHOX22 under various abiotic stress conditions were analyzed using publicly available microarray data from Genevestigator, which comprised of expression profiling data in 7-day-old rice (IR64) seedlings subjected to 3 h of desiccation, salinity and cold stresses, in drought-tolerant rice seedlings (N22) under drought stress, and in flag-leaf tissues of two rice genotypes; IRAT109 (drought-resistant *japonica* cultivar) and Zhenshan 97 (ZS97; drought sensitive *indica* cultivar) under drought stress at reproductive stage of development. The transcript levels of most of these genes were found to be altered under atleast one or more of the abiotic stress conditions analyzed (**Figure 6C**). The genes encoding for sucrose synthase, hypoxia-responsive family protein and GRAM domain TF showed similar expression profiles as that of *OsHOX24* and *OsHOX22* under selected abiotic stress conditions analyzed in Genevestigator (**Figure 6C**).

### Generation of *OsHOX24* Over-expression Transgenic *Arabidopsis* Plants

**FIGURE 7:**
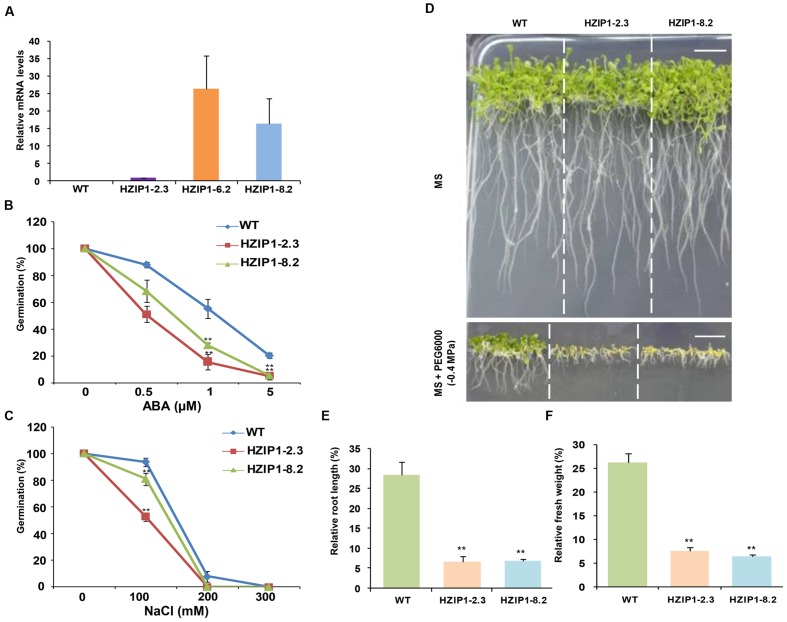
***Arabidopsis* transgenics (*35S::OsHOX24*) show higher sensitivity to abiotic stresses as compared to WT plants at seedling stage.** **(A)** Real-time PCR analysis of total RNA isolated from 12-day-old WT and homozygous *35S*::*OsHOX24* transgenic *Arabidopsis* seedlings (HZIP1-2.3, HZIP1-6.2, and HZIP1-8.2) under control conditions. The expression levels in transgenic lines were calculated relative to the transgenic line (HZIP1-2.3) exhibiting lowest transgene expression. Values are mean from three biological replicates. **(B,C)** Effect of ABA **(B)** and NaCl **(C)** on seed germination of WT and *35S::OsHOX24* (HZIP1-2.3 and HZIP1-8.2) *Arabidopsis* transgenic lines. The number of germinated seeds was expressed as the percentage of total number (40–60) of seeds plated. Values are mean from three independent experiments. **(D)** Phenotype of transgenic and WT *Arabidopsis* seedlings grown on MS media without and with PEG6000 at the seedling stage (scale bar = 1 cm). **(E,F)** Effect of PEG6000 (-0.4 MPa) treatment on root length **(E)** and fresh weight **(F)** of *35S::OsHOX24* transgenic lines as compared to WT *Arabidopsis* plants after 10 days of stress. Experiments were performed in three independent biological replicates. Values are mean (*N* = 12) from a single representative biological replicate. Error bars indicate SE. Bars marked with asterisk indicate statistically significant (^∗∗^*P*-value ≤ 0.001) difference between WT and transgenic lines.

The relatedness of *OsHOX22* and *OsHOX24* has been speculated to be a result of ancient chromosomal duplication (Agalou et al., 2008; Jain et al., 2008). Since these genes are expected to have redundant functions, we carried out functional characterization of *OsHOX24* in *Arabidopsis*. The complete ORF of *OsHOX24* was cloned in binary vector pBI121 and over-expressed under the control of CaMV 35S promoter in *Arabidopsis* (**Supplementary Figure S5A**). A total of 29 independently transformed kanamycin-resistant T1 transgenic plants for 35S::*OsHOX24* were obtained. Among them, a total of 19 T1 transgenic lines of 35S::*OsHOX24* were found to be PCR positive (**Supplementary Figure S5B**). Three transgenic lines (designated as HZIP1-2.3, HZIP1-6.2, and HZIP1-8.2), showing segregation ratio of nearly 3:1 were grown further to obtain homozygous seeds for physiological and molecular analysis. The real-time PCR analysis showed very high transcript levels of *OsHOX24* in homozygous transgenic lines, whereas it was not detectable in WT seedlings (**Figure 7A**). Among all 35S::*OsHOX24* homozygous transgenic lines, maximum expression was observed in HZIP1-6.2 line followed by HZIP1-8.2 (**Figure 7A**). There was no detectable difference in the phenotype and various growth parameters of *OsHOX24* transgenics as compared to WT at different developmental stages under normal growth conditions (**Supplementary Figures S6** and **S7**).

### *Arabidopsis* Transgenics Show Greater Sensitivity to Abiotic Stresses

The effect of plant stress hormone, ABA, and salinity stress on 35S::*OsHOX24* (HZIP1-2.3 and HZIP1-8.2) transgenic lines and WT was assessed via seed germination assays. The percentage germination of transgenic lines was observed to be much lesser as compared to WT on MS medium supplemented with various concentrations of ABA (0.5, 1, and 5 μM) and NaCl (100, 200, and 300 mM). The effect of ABA was more severe on the seed germination and growth of transgenics as compared to WT. For instance, at 0.5 μM ABA, WT showed 87% seed germination, whereas transgenics showed 51–68% germination. In presence of 1 μM ABA, WT exhibited 55% seed germination in comparison to 15–28% seed germination in the transgenics. Seed germination in transgenic lines was further reduced to 5% at 5 μM ABA, whereas WT seeds displayed 20% germination (**Figure 7B**). A greater extent of susceptibility in transgenics was observed as compared to WT under salinity stress too. The WT seedlings were relatively healthier and showed 93% germination, whereas the transgenic lines exhibited 52–80% germination at 100 mM NaCl. At 200 mM NaCl, 7% of WT seedlings germinated as compared to no germination of the transgenics (**Figure 7C**). Among the two 35S::*OsHOX24* lines, HZIP1-2.3 showed higher sensitivity to ABA and NaCl.

**FIGURE 8:**
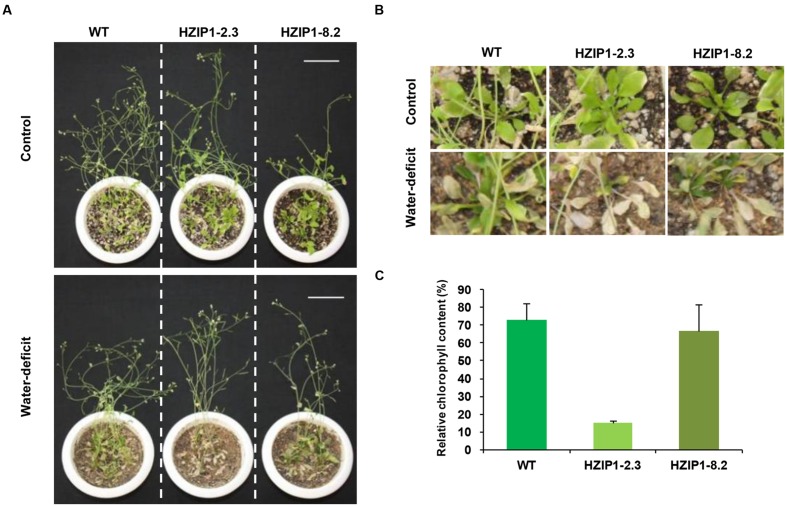
**Mature *35S::OsHOX24 Arabidopsis* transgenics are susceptible to water-deficit stress.** **(A)** Phenotype of *35S*::*OsHOX24* transgenics (HZIP1-2.3 and HZIP1-8.2) and WT *Arabidopsis* plants at mature stage after 3 weeks of water-deficit stress followed by recovery is shown (scale bar = 5 cm). **(B)** Effect of water-deficit stress on rosette leaves and growth of *Arabidopsis* transgenic lines as compared to WT [Enlarged image of **A**]. **(C)** The relative chlorophyll content in rosette leaves of plants under water-deficit condition was expressed as percentage of chlorophyll content in leaves of plant under control conditions. The experiments were performed in at least three independent biological replicates. Values shown are mean (*N* = 3) from a single biological replicate. Error bars indicate SE.

To evaluate the effect of desiccation stress on 35S::*OsHOX24* transgenic lines as compared to WT, relative fresh weight and root length of seedlings were estimated under desiccation stress (-0.4 MPa PEG6000) and control conditions. A significant difference in the phenotype of transgenics and WT seedlings was observed under desiccation stress (**Figure 7D**). The transgenic lines exhibited 5–6% of relative root length under desiccation stress (PEG) in comparison to 28% in WT (**Figure 7E**). Similarly, transgenic lines had significantly lesser fresh weight as compared to WT in the presence of PEG. Notably, transgenics lines exhibited only 3–7% of relative fresh weight under desiccation stress in comparison to WT, which showed 26% of relative fresh weight (**Figure 7F**). Four-week-old transgenic lines of 35S::*OsHOX24* subjected to water-deficit stress wilted at a faster rate than WT (**Figures 8A,B**). The extent of chlorosis was more prominent in the rosette leaves of transgenics as compared to WT (**Figure 8C**). Overall, these observations indicated that 35S::*OsHOX24* transgenics are more susceptible to water-deficit stress as compared to WT at mature stage too. We observed slightly greater susceptibility of HZIP1-2.3 line as compared to HZIP1-8.2 line during seed germination under ABA and salinity stress treatments. However, HZIP1-8.2 line exhibited significantly lesser sensitivity toward water-deficit stress as compared to HZIP1-2.3 line. The variation in the extent of susceptibility between the two transgenic lines may be attributed to the developmental stage and/or stress-type.

### Global Gene Expression Profiling of *OsHOX24 Arabidopsis* Transgenics

**FIGURE 9:**
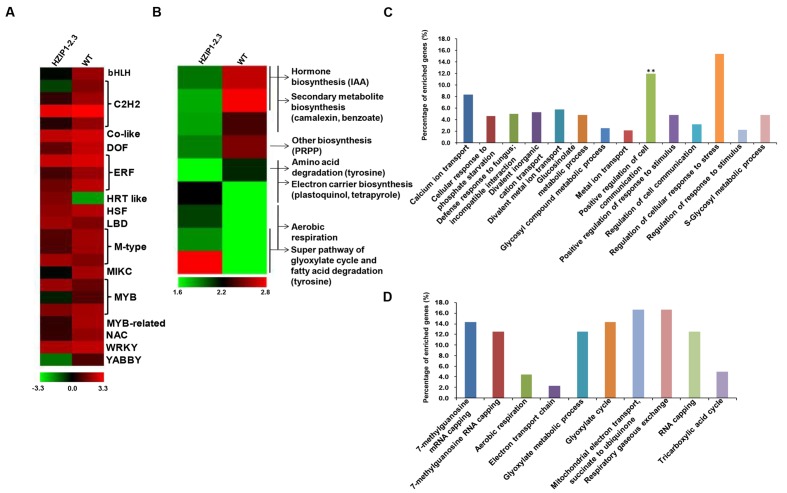
**Differential gene expression in *35S*::*OsHOX24* transgenic plants.** **(A,B)** Heatmaps representing expression profiles of all the differentially expressed TFs **(A)** and genes involved in diverse metabolic pathways **(B)** in the transgenic line as compared to WT are shown. The color scale representing average log signal values is shown at the bottom. **(C,D)** GO (biological process) enrichment in differentially expressed genes in the transgenics. Significantly enriched GO terms (*P-*value ≤ 0.05) in biological process category among down-regulated **(C)** and up-regulated **(D)** genes are shown. The bar marked with asterisk indicate terms with high statistical significance (*^∗∗^P*-value ≤ 0.001).

To examine the effect of *OsHOX24* over-expression on global gene expression, HZIP1-2.3 transgenic line (exhibiting relatively higher susceptibility to various abiotic stresses) was chosen for microarray analysis. A total of 292 genes (112 up-regulated and 180 down-regulated) were found to be significantly (at least twofold, *P* ≤ 0.05) differentially regulated in the transgenic line as compared to WT (**Supplementary Figure S8**; **Supplementary Table S4**). About 8% of the differentially expressed genes belonged to TF category (**Figure 9A**) and many of them were well known to be stress-responsive. In addition, pathway analysis depicted the involvement of differentially expressed genes in diverse metabolic pathways and developmental processes, such as hormone biosynthesis, secondary metabolite biosynthesis, electron carrier biosynthesis, amino acid, and fatty acid degradation pathways (**Figure 9B**).

Gene Ontology enrichment analysis revealed the differential regulation of genes involved in several biological processes in the transgenic line. The biological process GO terms, such as regulation of cellular response to stress and positive regulation of cell communication, showed highest representation among the down-regulated genes (**Figure 9C**), whereas mitochondrial electron transport, respiratory gaseous exchange, mRNA capping, and glyoxylate cycle were most represented GO terms among the up-regulated genes (**Figure 9D**). In the molecular function category, calcium-transporting ATPase activity, TF activity, binding, and several enzymatic activities were most significantly enriched among the down-regulated genes (**Supplementary Figure S9A**), whereas some vital enzymatic activity terms showed higher representation among the up-regulated genes (**Supplementary Figure S9B**).

Further, differential expression patterns of selected stress-inducible *Arabidopsis* genes, such as genes encoding VQ motif protein (*AT4G20000*), ribosomal binding protein L12 (*AT2G03130*), AP2 TF (*AT2G20880*), thioredoxin (*AT1G69880*), salt tolerance zinc finger (*AT1G27730*), and lipid binding protein (*AT5G59310*), were validated by real-time PCR analysis. The transcript profiling of these genes revealed their down-regulation in the transgenic line as compared to WT (**Supplementary Figure S10**), which was in good agreement with the microarray results.

## Discussion

Homeobox TFs are among the key regulators of plant development (Ariel et al., 2007). However, their role in abiotic stress responses in plants has been realized only in the past few years (Olsson et al., 2004; Luo et al., 2005; Agalou et al., 2008; Jain et al., 2008; Song et al., 2012). Earlier, we reported the differential expression of at least 37 homeobox genes under various abiotic stress conditions, many of which belong to the plant-specific HD-ZIP superclass (Jain et al., 2008). A few other studies have also reported differential regulation of HD-ZIP class homeobox genes under abiotic stress conditions (Gago et al., 2002; Olsson et al., 2004; Agalou et al., 2008; Bhattacharjee et al., 2015). About 64% of rice HD-ZIP I subfamily members were found to be abiotic stress-responsive (Jain et al., 2008).

The present study was focused on the molecular characterization and functional analysis of two candidate abiotic stress-responsive homeobox genes, *OsHOX24* and *OsHOX22*. These genes were found to be highly up-regulated under different abiotic stress conditions at various developmental stages in rice. In earlier studies, *OsHOX24* and *OsHOX22* were reported to be highly expressed under control and drought stress in panicle at the flowering stage of rice (Agalou et al., 2008; Jain et al., 2008), which is consistent with our observations. These results implicate *OsHOX24* and *OsHOX22* in abiotic stress responses at various developmental stages of rice. The role of major *cis*-regulatory elements, like DRE, ABRE, MYB recognition sequence (MYBR), NAC recognition sequence (NACRS), Heat shock element (HSE), and ZF-HD recognition sequence (ZFHDRS), etc. in abiotic stress responses have been investigated comprehensively (Stockinger et al., 1997; Hobo et al., 1999; Dezar et al., 2005; Liu et al., 2014). We found enrichment of numerous stress-responsive *cis*-regulatory elements, like ABRE, CBF-DRE, LTRE, and MYB elements in the promoters of *OsHOX24* and *OsHOX22*. The presence of these *cis*-regulatory elements in their promoter regions may contribute to their abiotic stress-responsiveness. Recently, the use of drought-responsive promoter of a rice HD-ZIP I gene, enriched in various stress-responsive *cis-*regulatory elements, was found to be beneficial for over-expression of specific stress-responsive genes without any detrimental effect on plant growth (Nakashima et al., 2014).

Various plant hormones play critical roles in abiotic stress responses (Wang et al., 2002; Horvath et al., 2007; Grant and Jones, 2009; Jain and Khurana, 2009; Tran et al., 2010; Fujita et al., 2011; Sharma et al., 2015). Several evidences have demonstrated the interrelation between plant hormones and homeobox TFs. *Arabidopsis ATHB7* and *ATHB12*, orthologs of *OsHOX24* and *OsHOX22*, respectively, were found to be highly induced on exogenous application of ABA, indicating their involvement in ABA-dependent pathways (Olsson et al., 2004). Recently, these TFs were found to actively participate in ABA signaling by controlling protein phosphatase 2C and ABA receptor gene activity (Valdés et al., 2012). The members of HD-ZIP superclass have also been reported to be involved in gibberellin and auxin signaling (Dai et al., 2008; Itoh et al., 2008; Sharma et al., 2015). We also observed the up-regulation of *OsHOX24* and *OsHOX22* by exogenous application of ABA, IAA, and SA. These results suggest the involvement of homeobox genes in ABA, IAA, or SA-dependent stress response pathways in rice. However, their exact role in various hormone signaling pathways remains to be elucidated.

Both OsHOX24 and OsHOX22 comprise of conserved HD and HALZ domains. The high degree of structural conservation in three-dimensional HD structures of OsHOX24 and OsHOX22 with antennapedia-HD DNA complex of *Drosophila* (Fraenkel and Pabo, 1998) suggested that they are likely to possess DNA binding property. Some reports have demonstrated DNA binding specificities of homeobox proteins with specific pseudopalindromic sequences *in vitro* (Sessa et al., 1997; Frank et al., 1998). Using specific recognition sites, namely AH1 and AH2, the binding specificities of HD-ZIP I TFs have been examined in rice and *Arabidopsis* (Meijer et al., 2000; Johannesson et al., 2001; Zhang et al., 2012). In this study, we also demonstrated the binding specificity of OsHOX24 and OsHOX22 proteins with AH1 and AH2 motifs. These observations imply that the binding specificities of homeobox proteins are conserved in different plants.

One of the possible modes of action of homeobox TFs in abiotic stress response may be via regulation of downstream target genes. However, limited information is available about their downstream target genes. A dehydrin gene, *CdeT6-19*, has been identified as potential target of *CpHB-7* (Deng et al., 2006). The genes involved in ethylene synthesis and signaling were found to be downstream targets of Hahb-4 (Manavella et al., 2006). A genome-wide scan identified at least 809 rice genes harboring AH1 and/or AH2 motifs in their promoter regions which might represent their putative downstream target genes. Many of these genes are involved in crucial biological and developmental processes. These results suggest that OsHOX24/OsHOX22 TFs may regulate the expression of downstream target genes involved in diverse biological processes to mediate abiotic stress responses.

Several studies have demonstrated the transactivation property in TFs, due to the presence of intrinsic activation domain (Lu et al., 2009; Tang et al., 2012; Yang et al., 2014). Particularly, carboxy-terminal region of AtHB1 was identified to be responsible for transcriptional activation property in yeast (Arce et al., 2011). Among rice HD-ZIP TFs, OsHOX1 and OsHOX3 exhibited transcriptional repression activity, whereas OsHOX4 and OsHOX5 were recognized as transcriptional activators (Meijer et al., 2000). We noted the presence of an activation domain in the C-terminal region of OsHOX24/OsHOX22 proteins, which imparted transactivating nature to these proteins. We found OsHOX24/OsHOX22 proteins to be localized in the nucleus, consistent with the presence of a NLS in their amino acid sequence and with earlier reports for other homeobox TFs to be nuclear-localized (Song et al., 2012; Zhang et al., 2012). Altogether, these evidences suggest that OsHOX24/OsHOX22 are nuclear-localized and can function as transcriptional activators.

The current knowledge about interacting proteins of homeobox TFs is limiting. We identified several proteins, including enzymes, receptor protein, expressed proteins, and a TF as putative interacting proteins of OsHOX24 and OsHOX22. Many of these putative interacting proteins were found to be abiotic stress-responsive. There are several instances, where interaction between two TFs has been found to crucially mediate abiotic stress responses in plants (Tran et al., 2007; Lee et al., 2010). We identified GRAM domain TF as putative interacting protein of OsHOX24. Interestingly, lower transcript levels of GRAM domain TF-gene was detected in ABA-sensitive *Osabf1* rice mutants (Amir Hossain et al., 2010). It is well established that sucrose metabolism is severely affected by environmental alterations, which leads to strong impact on plant development (Koch, 2004). Many genes including members of sucrose synthase family and a UDP-glucosyltransferase gene have been implicated in abiotic stress responses in plants (Gupta and Kaur, 2005; Hirose et al., 2008; Tognetti et al., 2010; Wang et al., 2015). Very recently, OsPAL4 (phenylalanine ammonia lyase) has been implicated in broad spectrum disease resistance (Tonnessen et al., 2015). In our investigation, sucrose synthase (SUS4) and phenylalanine lyase were identified as the interacting partners of OsHOX24, whereas UDP-glucuronosyl and UDP-glucosyl transferase domain containing protein was found to be interacting partner of OsHOX22 in yeast. A translation initiation factor has been reported to elicit abiotic stress tolerance in yeast and plants (Rausell et al., 2003). Interestingly, we too found eukaryotic translation initiation factor I to be an interactor of OsHOX24. This interaction may be crucial for regulation of translation initiation under abiotic stress conditions. Recently, a universal stress protein was found to accentuate drought tolerance in tomato (Loukehaich et al., 2012). We also identified an universal stress protein domain containing protein as one of the interacting proteins of OsHOX22. These observations indicated that homeobox TFs interact with other proteins to modulate abiotic stress responses.

The analysis of few mutant and transgenic lines of homeobox TFs has revealed their role in abiotic stress responses in plants (Olsson et al., 2004; Zhu et al., 2004; Luo et al., 2005; Yu et al., 2008; Zhang et al., 2012). Recently, the overlapping and explicit roles of *ATHB7* and *ATHB12* in modulating various aspects of plant development and responses to water-deficit stress have been delineated (Ré et al., 2014). In a previous study, rice BELL-type homeobox TF, *OsB1HD1*, has been reported to act as a negative regulator by suppressing the abiotic stress signaling cascade in over-expression tobacco transgenic lines (Luo et al., 2005). The role of *OsHOX22* has been deciphered in ABA-dependent abiotic stress responses, which was also found to act as negative regulator of drought and salt tolerance in rice (Zhang et al., 2012). We over-expressed *OsHOX24* in *Arabidopsis* to substantiate its role in abiotic stress responses, and analyzed drought and salinity stress responses of WT and transgenic plants at various developmental stages. These studies revealed higher susceptibility of transgenics as compared to WT under abiotic stress conditions. Several reports have implicated plant hormone ABA in abiotic stress responses (Cutler et al., 2010; Fujita et al., 2011). We observed enhanced sensitivity of *OsHOX24 Arabidopsis* transgenics under exogenous ABA treatment. Previous investigations have also demonstrated the ABA-inducible nature of HD-ZIP I family members in model plants and established their role in ABA signaling (Olsson et al., 2004; Valdés et al., 2012; Zhang et al., 2012). Overall, our results in conjunction with available reports suggest that *OsHOX24* and *OsHOX22* may act in an ABA-dependent abiotic stress response pathway.

Several genes were found to be differentially expressed in *OsHOX24 Arabidopsis* transgenics, which were related to secondary metabolite biosynthesis, electron carriers and IAA biosynthesis, and amino acid and fatty acid degradation pathways. The role of secondary metabolites, amino acids, fatty acids, and electron carriers are well known in plant stress adaptation (Ramakrishna and Ravishankar, 2011; El-kereamy et al., 2012; Anjum et al., 2015; Kapoor, 2015). Besides this, the crucial role of IAA in abiotic stress responses has also been proposed (Jain and Khurana, 2009; Sharma et al., 2015). Down-regulation of genes involved in biosynthesis of osmoprotectants or secondary metabolites, coupled with elevated transcript levels of genes involved in fatty acid degradation may be responsible for greater susceptibility of *Arabidopsis* transgenics to abiotic stresses. Several stress-responsive genes are known to be induced under various abiotic stress conditions in plants (Walley et al., 2007; Pitzschke et al., 2009; Kim et al., 2010). The down-regulation of these genes in the *Arabidopsis* transgenics can explain their higher susceptibility to various abiotic stresses to some extent.

## Conclusion

*OsHOX24* and *OsHOX22* were found to be differentially expressed under various abiotic stress conditions at different stages of rice development. We demonstrated that these nuclear-localized homeobox TFs possess transactivation and dimerization properties. We also identified novel interacting proteins of homeobox TFs and many of them are stress-responsive. We showed the binding ability of OsHOX22 and OsHOX24 with specific *cis*-regulatory elements and identified several putative downstream targets. The over-expression of *OsHOX24* imparted higher susceptibility to various abiotic stresses in the transgenic *Arabidopsis* plants as revealed by several physiological and molecular assays. Overall, our results highlight the role of OsHOX24 and OsHOX22 TFs in abiotic stress responses. In future, the generation and analysis of knock-out transgenic lines would be able to provide more insights about the role of these homeobox TFs in abiotic stress tolerance.

## Author Contributions

MJ conceived and supervised the whole study. AB performed all the experiments, analyzed the data and wrote the manuscript. MJ and JPK participated in data analysis and writing the manuscript.

## Conflict of Interest Statement

The authors declare that the research was conducted in the absence of any commercial or financial relationships that could be construed as a potential conflict of interest.

## Footnotes

1 http://www.dna.affrc.go.jp/PLACE/signalscan.html

2 http://mordred.bioc.cam.ac.uk/\tilderapper/rampage.php

3 https://genevestigator.com/gv/
