# Supplementary material for: Characterization of Rice Homeobox Genes, OsHOX22 and OsHOX24, and Over-expression of OsHOX24 in Transgenic Arabidopsis Suggest Their Role in Abiotic Stress Response
Source: Front Plant Sci. 2016 May 10;7:627. doi: 10.3389/fpls.2016.00627 (PMC4862318; doi:10.3389/fpls.2016.00627)
Supplement: Supplementary file 1 [file Table_1.PDF]

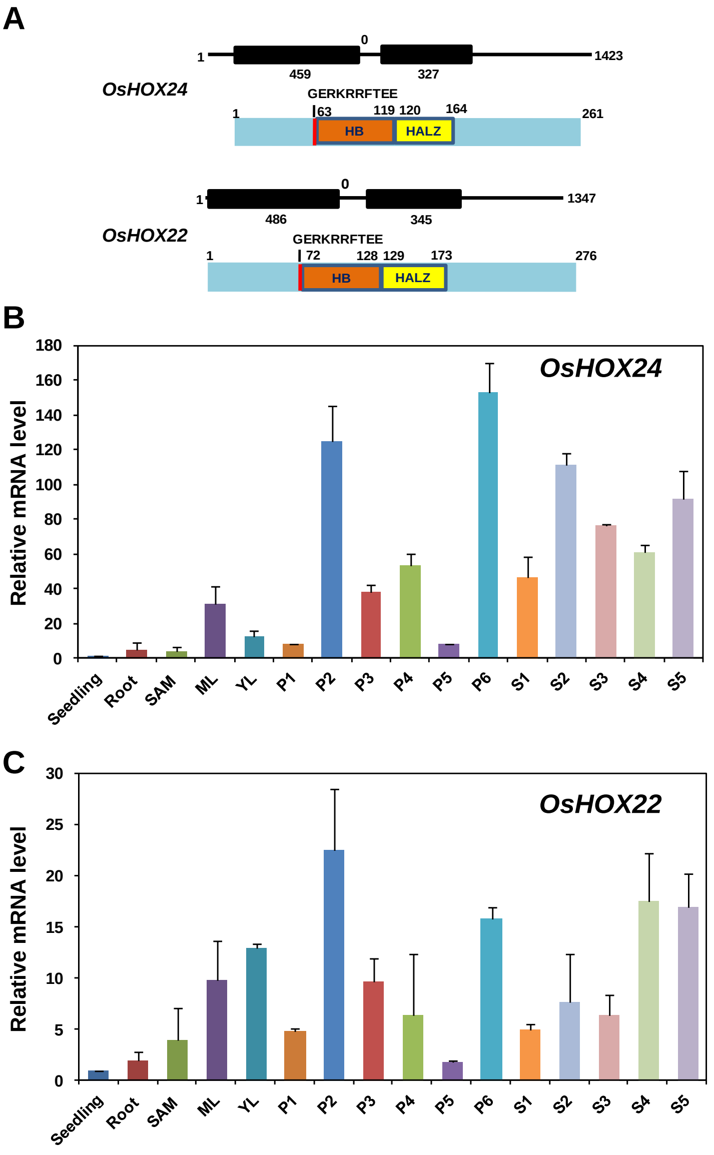

FIGURE S1 | Gene structure, domain organization and expression profiles of rice homeobox genes. (A) The upper panel shows exon-intron organization of *OsHOX24* and *OsHOX22* genes. Numbers above the vertical bars represent the phase 0 intron. The homeobox proteins contain a homeobox (HB) domain and a homeobox associated leucine zipper (HALZ) region as shown in lower panel. The amino acid sequence above the homeodomain signifies the nuclear localization signal. **(B, C)** The relative mRNA levels of *OsHOX24* **(B)** and *OsHOX22* **(C)** genes during various stages of development in rice as revealed by real-time PCR analysis. The mRNA levels for each gene in different tissue samples were calculated relative to its expression in the seedling. SAM, shoot apical meristem; ML, mature leaf; YL, Y leaf; P1 to P6, stages of panicle development (P1, 0-3 cm; P2, 3-5 cm; P3, 5-10 cm; P4, 10-15 cm; P5, 15-22 cm; P6, 22-30 cm); and S1 to S5, stages of seed development (S1, 0-2 DAP {Days after pollination}; S2, 3-4 DAP; S3, 5-10 DAP; S4, 11-20 DAP; S5, 21-29 DAP). Values are mean ± SE (N=2) from two representative biological replicates. Error bars indicate standard error.

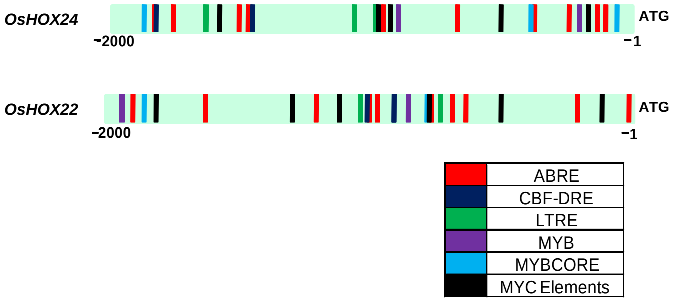

FIGURE S2 | Schematic representation of 2 kb promoter region of *OsHOX24* and *OsHOX22*. The *cis*-regulatory stress-responsive motifs present in the promoter regions of *OsHOX24* and *OsHOX22* are shown in different colors.

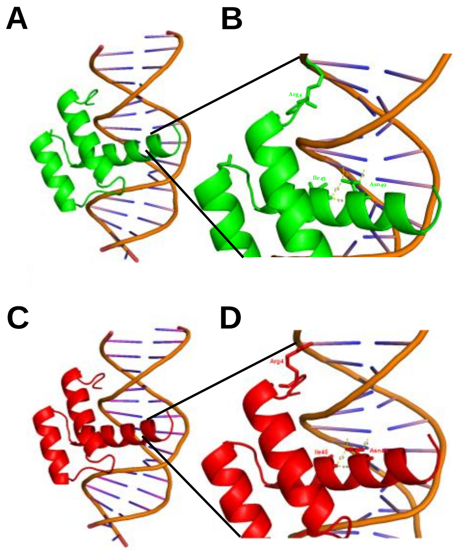

FIGURE S3 | Three-dimensional structure of homeodomains of OsHOX24 and OsHOX22 proteins and their binding with DNA. (A, C) Association of homeobox domain of OsHOX24 **(A)** and OsHOX22 **(C)** with DNA. **(B, D)** Hydrogen bonding between homeobox domain of OsHOX24 **(B)** and OsHOX22 **(D)** with major groove of DNA.

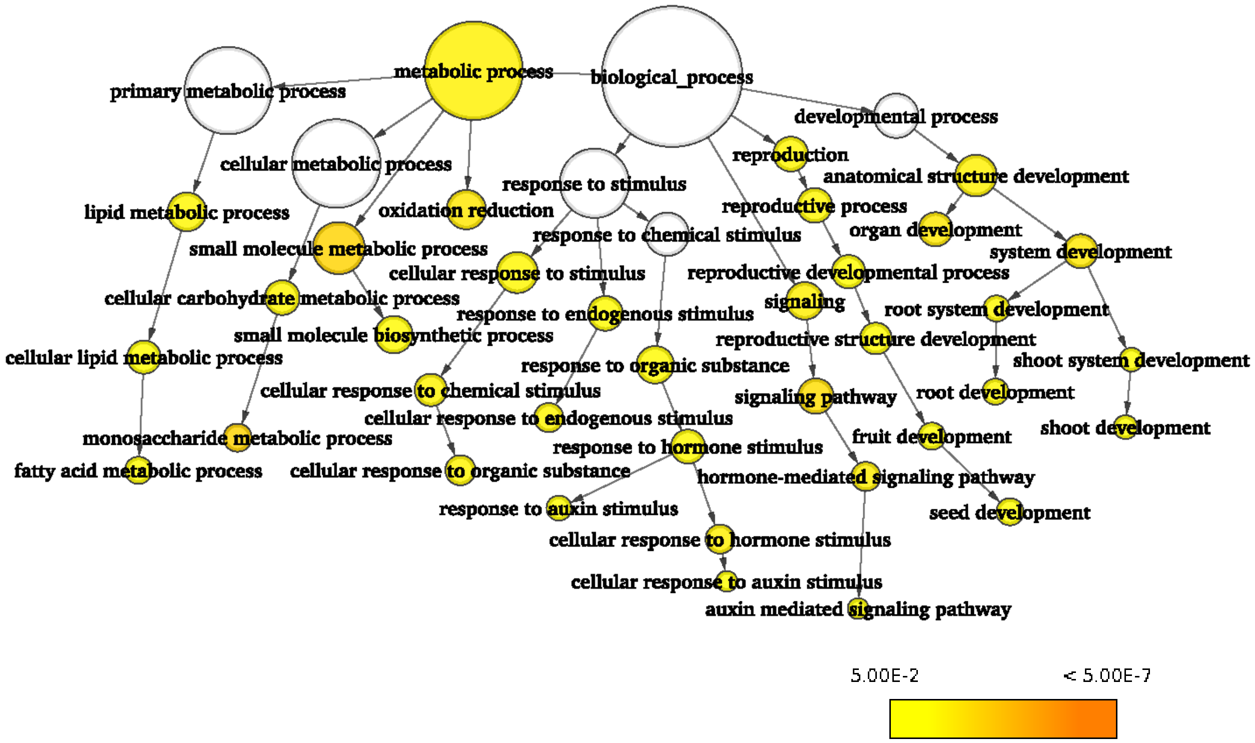

FIGURE S4 | Gene ontology (GO) terms found enriched among the rice genes harbouring AH1 and/or AH2 motifs in their 1 kb promoter region. GO terms in biological process category among rice genes (harbouring AH1 and/or AH2 motifs in 1 kb promoter region) were analyzed using BiNGO and the terms showing significant enrichment have been highlighted. Node size is proportional to the number of genes in each category and color shading is given according to the significance level (white-no significant difference; color scale, yellow-P-value = 0.05, orange-P-value < 0.0000005).

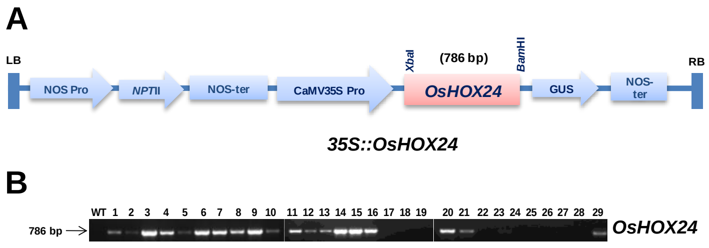

FIGURE S5 | Cloning of *OsHOX24* ORF in pBI121 binary vector and confirmation of *Arabidopsis* transgenics by PCR. (A) Schematic representation of over-expression construct of *OsHOX24* used for raising transgenics in *Arabidopsis*. **(B)** PCR confirmation of putative *35S::OsHOX24 Arabidopsis* transgenic plants. A total of 19 plants were found to be PCR positive from 29 kanamycin-positive plants obtained for *OsHOX24*.

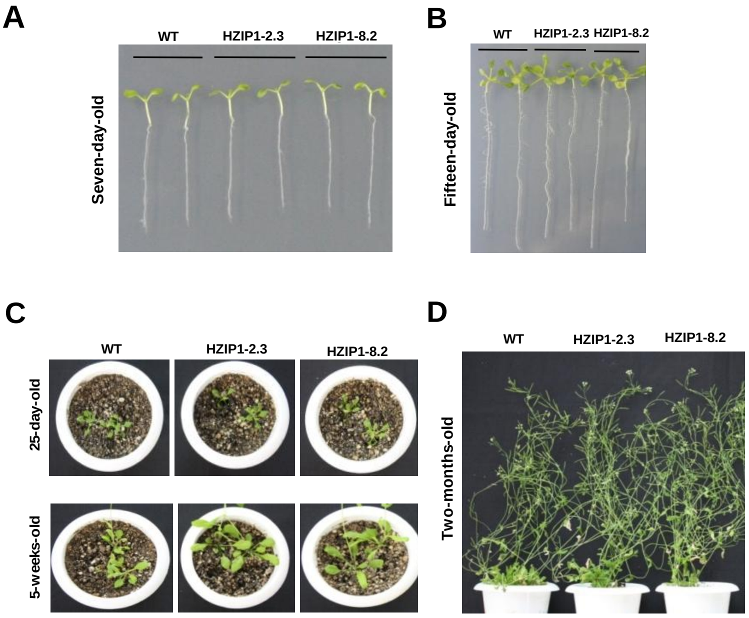

FIGURE S6 | Phenotype of *35S::OsHOX24* and WT *Arabidopsis* (Col-0) plants at different developmental stages. (A) Seven-day-old seedlings on MS agar, **(B)** Fifteen-day-old seedlings on MS agar, **(C)** Rosette at 25-day-old stage and five-week-old plants grown in soilrite, **(D)** Two-month-old mature plants with fully developed siliques.

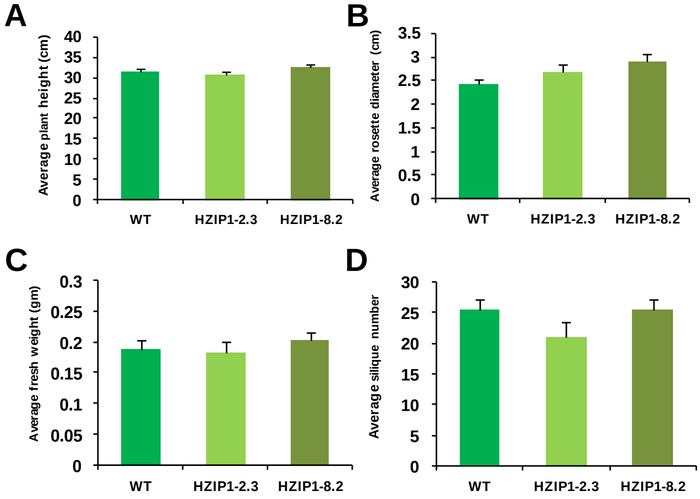

FIGURE S7 | Growth parameters of mature *35S::OsHOX24* and wild-type (WT) *Arabidopsis* plants under control conditions. Various growth parameters, like average plant height **(A)**, rosette diameter **(B)**, fresh weight **(C)** and silique number **(D)** of *35S::OsHOX24* transgenic lines (HZIP1-2.3 and HZIP1-8.2) and WT *Arabidopsis* plants after two months of growth have been represented. The measurement of growth parameters of WT and *35S::OsHOX24 Arabidopsis* transgenics have been done in atleast three independent biological replicates. Values shown are mean ± SE from one representative biological replicate (N=20).

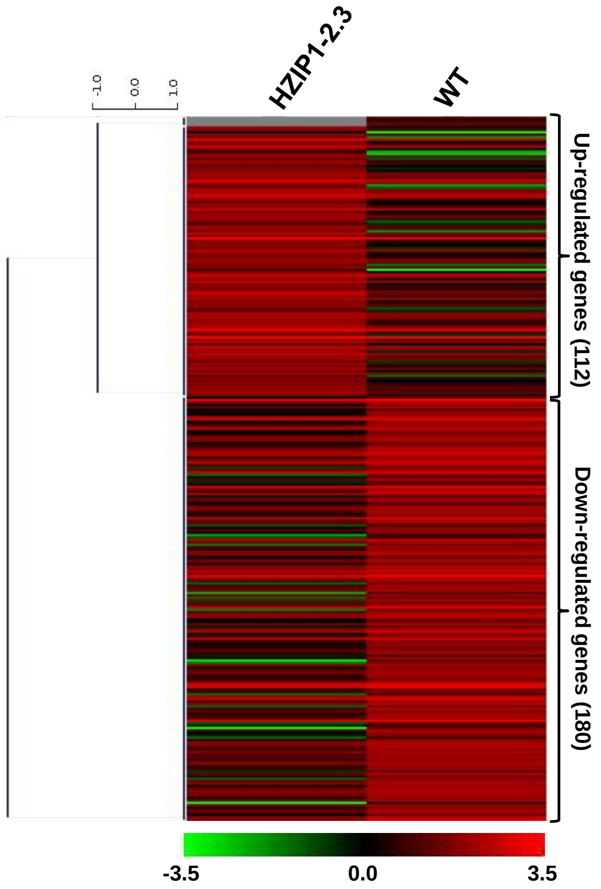

FIGURE S8 | Differential gene expression in 35S::*OsHOX24* transgenic line (HZIP1-2.3) as compared to wild-type (WT) *Arabidopsis*. Heatmap represents expression profile of 292 differentially expressed (112 upregulated and 180 downregulated) genes in the transgenic line as compared to WT. The color scale (representing average log signal values) is shown below the heatmap.

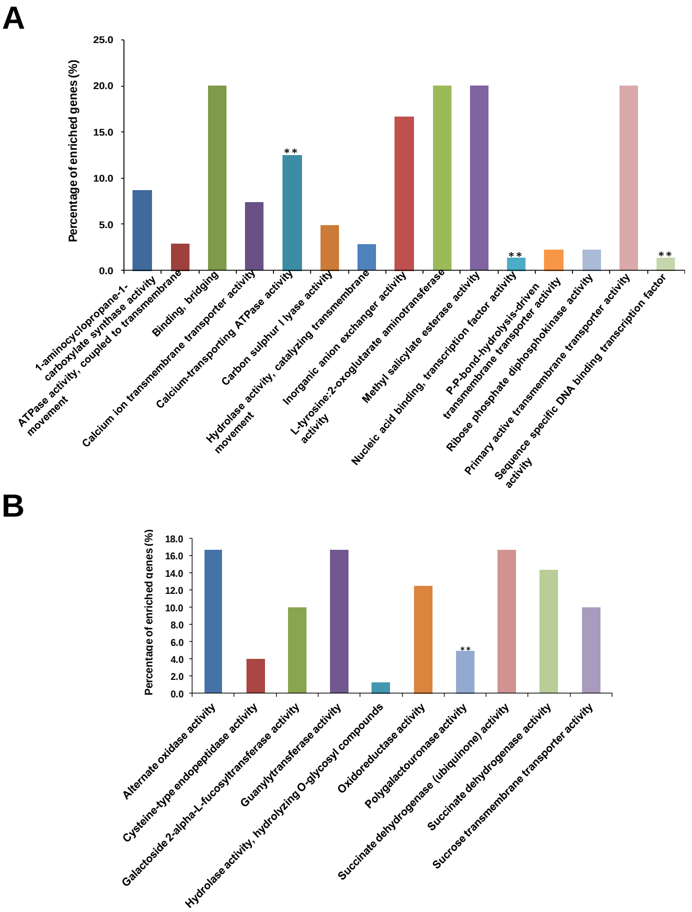

FIGURE S9 | Gene ontology (GO) (molecular function) enrichment in differentially expressed genes in 35S::*OsHOX24* transgenic *Arabidopsis* seedlings. (A, B) Significantly enriched GO terms (*P*-value ≤ 0.05) found in molecular function category among downregulated **(A)** and upregulated **(B)** genes in the transgenic line as compared to wild-type. The bars marked with "**" asterisk indicate GO terms with high statistical significance (*P*-value ≤ 0.001).

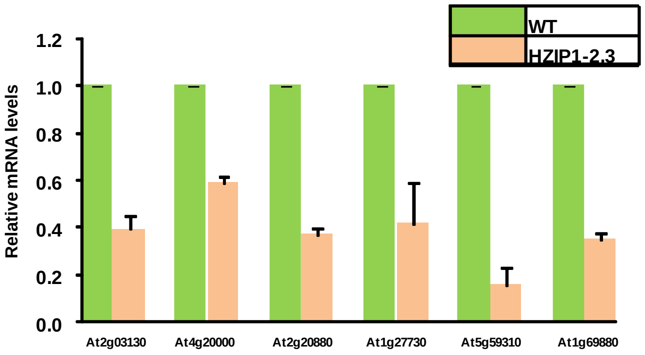

FIGURE S10 | Real-time PCR validation of microarray results. Expression profiles of selected *Arabidopsis* genes (from the microarray analysis results) in HZIP1-2.3 transgenic line as compared to wild-type plants under control conditions are shown.
